# Construction of performance score dynamic prediction system for clinical departments using explainable machine learning

**DOI:** 10.1007/s13755-025-00394-y

**Published:** 2025-12-02

**Authors:** Huashu Wen, Xiaohua Li, Haibo Zhang, Birong Wen, Yaying Ren, Xia Zhao

**Affiliations:** General Hospital of Southern Theater Command of PLA, Guangzhou, 510010 Guangdong China

**Keywords:** Clinical department, Performance data, Artificial intelligence, Prediction model, Web system, Explainable machine learning

## Abstract

**Background:**

Accurate evaluation of clinical departmental performance is essential for public hospital management. However, existing approaches primarily rely on static, retrospective annual assessments and lack interpretability, limiting their ability to support early intervention and informed decision-making. To address these gaps, the study aimed to present a dynamic framework for predicting annual departmental performance score based on real-world hospital data using explainable machine learning.

**Methods:**

Performance data between January 2023 to December 2024 was collected from the Hospital Information System (HIS). Six machine learning models, namely Linear Regression (LR), Decision Tree (DT), Random Forest (RF), Gradient Boosting, XGBoost, CatBoost, were trained to predict annual performance scores across three cumulative time windows (January–March, January–June, January–September) with traning set of 2023 and testing set of 2024. Model Evaluation was using metrics such as R^2^, Root Mean Squared Error (RMSE), Mean Absolute Error (MAE), and Mean Squared Error (MSE). To comprehensively enhance interpretability of dynamic model, SHapley Additive exPlanations (SHAP) analysis was applied to reveal key performance indicators across different time windows in predicting annual departmental performance. The web prediction System was developed using streamlit based on best-performing model.

**Results:**

A total of 648 records covering 24 months was obtained from 27 clinical departments. Compared with among models, LR model achieved the highest R^2^ values (0.8964, 0.9314, 0.9640) and the lowest RMSE (0.0257, 0.0209, 0.0151), MAE (0.0190, 0.0162, 0.0116), and MSE (0.0007, 0.0004, 0.0002) across all stages. Through SHAP analysis, the top 5 contributing performance indicator were consistent across all stages, including proportion of medical service revenue in total medical revenue, proportion of outpatient revenue from medical insurance fund, proportion of consumables in total medical revenue, proportion of inpatient revenue from medical insurance fund, and average inpatient expense per admission. For clinical practice, the web tool, ClinDeptPredictor, was developed based on LR model, accesible at https://clindeptpredictor-tool.streamlit.app/.

**Conclusion:**

The study presented a novel dynamic framework for clinical departmental performance prediction in tertiary public hospital using explainable machine learning. The framework supports progressive performance monitoring across multiple time windows and provides timely insights to inform managerial decisions. In addition, the development of the web tool helps facilitate practical application, offering methodological support and practical reference for performance management in public healthcare settings.

## Introduction

The healthcare industry, both challenging and essential, currently faces several significant issues, including the escalating costs, inefficient service delivery, heightened industry competition, and the need for equity in service delivery and patient satisfaction, which are prevalent around the world [1–3]. Globally, 20–40% of healthcare resources are reportedly underutilized each year due to inefficiency [4, 5]. Hospitals, as the primary spenders in the healthcare system, account for 50–80% of the health sector budget in many countries [6–9]. Therefore, it is critical to assess hospital performance for effective management.

However, existing performance assessment practices largely rely on retrospective annual indicators or static dashboards derived from hospital information systems (HIS) and business intelligence tools [10–17]. These approaches often suffer from limited indicator coverage, delayed feedback, and insufficient support for proactive decision-making, particularly in complex tertiary public hospitals where operational dynamics can change rapidly. Recent advances in machine learning (ML) have shown strong potential for enhancing hospital operational management, such as scheduling optimization [18], capacity planning [19], resource allocation and inventory management [20], demand forecasting [21]. Despite these advancements, most studies focus on single operational tasks rather than providing a comprehensive evaluation framework at the clinical department level. Moreover, existing ML models typically rely on static, retrospective predictions with limited interpretability, which restricts their practicality for proactive hospital management.

To address these limitations, the study proposed a novel dynamic framework for predicting the annual performance of clinical departments using explainable ML technique. Specially, we applied six ML algorithms to build dynamic model, including Linear Regression [22], Decision Tree [23], Random Forest [24], Gradient Boosting [25], XGBoost [26], CatBoost [27]. The framework utilized cumulative monthly data to make progressive predictions across multiple temporal windows (January–March, January–June, January–September), enabling hospital managers to monitor performance trends and implement early interventions. Notably, performance indicators in the study were selected, guided by “National Tertiary Public Hospital Performance Assessment Manual (2024 Edition)” [28] and the actual operational characteristics of the hospital, ensuring alignment with operational practices and providing comprehensive coverage of financial performance, operational efficiency, and cost control. To enhance interpretability of dynamic model, SHAP analysis [29–31] was employed to quantify the contribution of individual indicators to the predicted scores.

Finally, a user-friendly web tool, ClinDeptPredictor, was developed using Streamlit [32–34] based on best-performing trained model to support real-world clinical management. Briefly, the system allowed users to input monthly departmental data, obtain dynamic predictions of annual performance scores and corresponding level, and visualize model interpretability through SHAP method, thereby supporting evidence-based managerial decisions and facilitating the optimization of hospital operations.

## Materials and methods

### Data collection & preprocessing

**Fig. 1:**
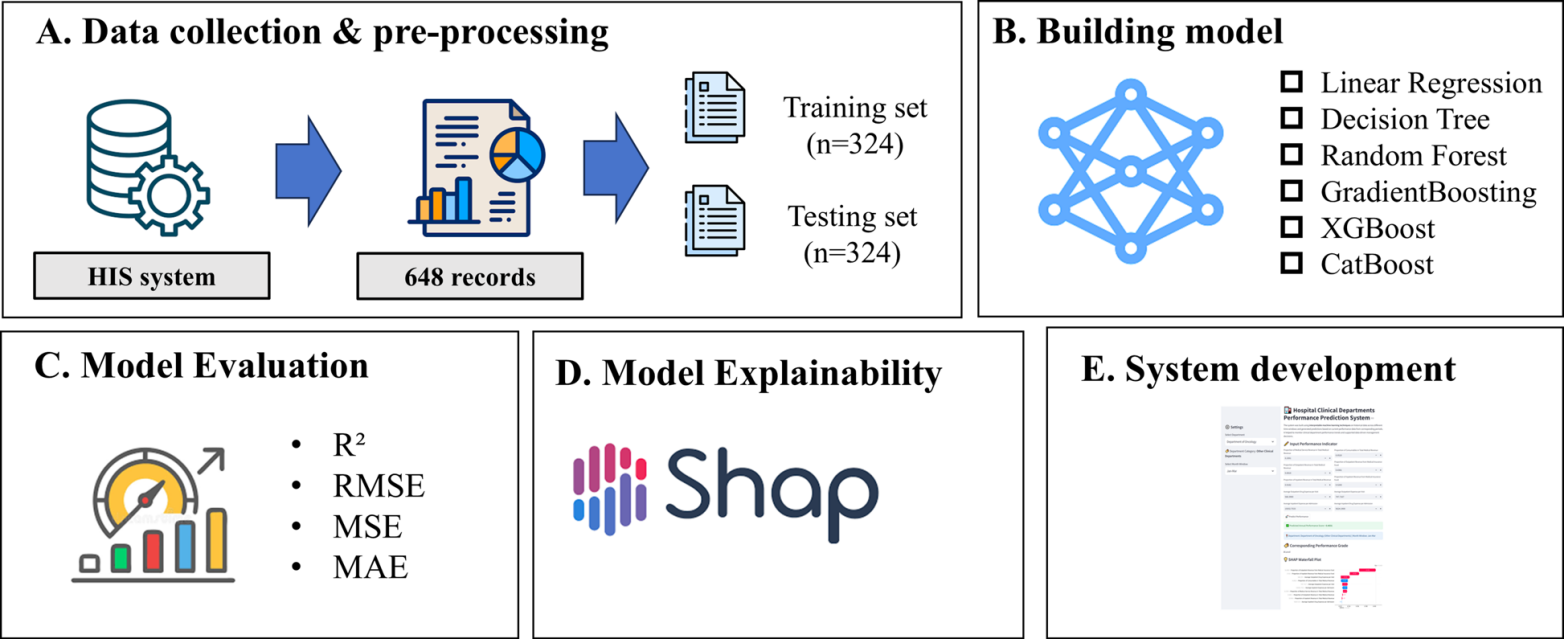
Overview of the study workflow

The study workflow was illustrated in Fig. 1. In this study, we collected performance data from 27 clinical departments between January 2023 and December 2024. A total of 648 records were extracted from the Hospital Information System (HIS) covering 24 months. Specially, the dataset consisted of ten feature variables (performance indicators) and a single target variable (annual performance score) (Table 1). To evaluate the model’s prospective performance, it was chronologically partitioned into a training set (*n* = 324, 2023) and a testing set (*n* = 324, 2024). The 27 clinical departments were categorized into four groups, namely Surgical Departments, Medical Departments, Departments of Obstetrics & Gynecology, and Pediatrics, Other Clinical Departments (Table 2). Based on the government policy guidelines document for tertiary public hospital performance evaluation [28] and the actual operational characteristics of the hospital, ten performance indicators were selected to assess departmental performance from two aspects, namely revenue and expenditure structure, and cost control (Table 1). The experiments were conducted in Python 3.10, with a fixed random seed (42).

**Table 1:** Performance indicators of clinical departments

Category	Indicators	Weight
Revenue & Expenditure Structure	Proportion of medical service revenue in total medical revenue	0.2
	Proportion of consumables in total medical revenue	0.05
	Proportion of outpatient revenue in total medical revenue	0.1
	Proportion of outpatient revenue from medical insurance fund	0.1
	Proportion of inpatient revenue in total medical revenue	0.1
	Proportion of inpatient revenue from medical insurance fund	0.1
Cost Control	Average outpatient drug expense per visit	0.05
	Average outpatient expense per visit	0.1
	Average inpatient expense per admission	0.15
	Average inpatient drug expense per admission	0.05

Indicator weights were assigned based on the government policy guidelines document and hospital operational practices

**Table 2:** List of clinical departments and departments categories in the study

Departments categories	Clinical departments
Medical departments	Department of respiratory medicine
	Department of endocrinology
	Department of general practice
	Department of neurology
	Department of nephrology
	Department of gastroenterology
	Department of cardiovascular medicine
	Department of hematology
Surgical departments	Department of otorhinolaryngology head & neck surgery
	Department of hepatobiliary surgery
	Department of orthopedics
	Department of urology
	Department of general surgery
	Department of burn and plastic surgery
	Department of neurosurgery
	Department of cardiothoracic surgery
	Department of cardiac surgery
Departments of obstetrics & gynecology, and pediatrics	Department of pediatrics
	Department of obstetrics and gynecology
Other Clinical Departments	Department of rheumatology and immunology
	Department of hyperbaric oxygen and rehabilitation
	Department of rehabilitation medicine
	Department of stomatology
	Department of dermatology
	Department of ophthalmology
	Department of traditional chinese medicine
	Department of oncology

### Calculation of annual performance score

The annual performance score for each clinical department was calculated by first normalizing the ten performance indicators within the same year using min–max scaling. The normalized indicators were then aggregated with pre-assigned indicator weights to obtain a monthly score (Table 1). Finally, the monthly scores were combined using month weights (Table 3) to calculate the final annual score, ensuring that each indicator and each month contributed appropriately to the overall departmental performance. The formula mentioned above is presented below:

$$\chi_{i,m}^{norm} \left( {\mathcal{Y}} \right) = \frac{{\chi_{i,m} \left( y \right) - \min_{m} \left( {\chi_{i,m} \left( y \right)} \right)}}{{\max_{m} \left( {\chi_{i,m} \left( y \right)} \right) - \min_{m} \left( {\chi_{i,m} \left( y \right)} \right)}}$$

where, $\chi_{i,m} \left( {\mathcal{Y}} \right)$ is the the raw value of indicator $i$ in month $m$ for year ${\mathcal{Y}}$, $\chi_{i,m}^{norm} \left( {\mathcal{Y}} \right)$ denote the min–max normalized value of indicator $i$ in month $m$ for that year.

$$S_{m} \left( {\mathcal{Y}} \right) = \sum_{i = 1}^{10} w_{i} \,\chi_{i,m}^{norm} \left( {\mathcal{Y}} \right)$$

where, $w_{i}$ denote the weight of indicator $i$.

$$Annual\, Performance \,Score \left( {\mathcal{Y}} \right) = \sum_{m = 1}^{12} V_{m} S_{m} \left( {\mathcal{Y}} \right)$$

where, ${\mathcal{V}}_{m}$ denote the weight of month $m$.

**Table 3:** Monthly weights for performance evaluation

Month	Weight	Month	Weight
January	0.05	July	0.05
February	0.05	August	0.05
March	0.1	September	0.1
April	0.1	October	0.1
May	0.1	November	0.1
June	0.1	December	0.1

Monthly weights was assigned, reflecting seasonal variations in clinical service demand (peak and off-peak periods) in line with hospital operational practices

## Model building & evaluation

The study employed six machine learning algorithms to build the model, including Linear Regression (LR), Decision Tree (DT), Random Forest (RF), Gradient Boosting, XGBoost, CatBoost. To achieve dynamic model prediction, three cumulative monthly windows (January–March, January–June, January–September) were designed for annual performance score prediction. Models were trained separately for each monthly window on training set. Subsequently, models evaluation were performed on testing set using metrics included R^2^, Root Mean Squared Error (RMSE), Mean Absolute Error (MAE), and Mean Squared Error (MSE). For each window, the model achieving the highest R^2^ was selected as the optimal model.

## System development

**Fig. 2:**
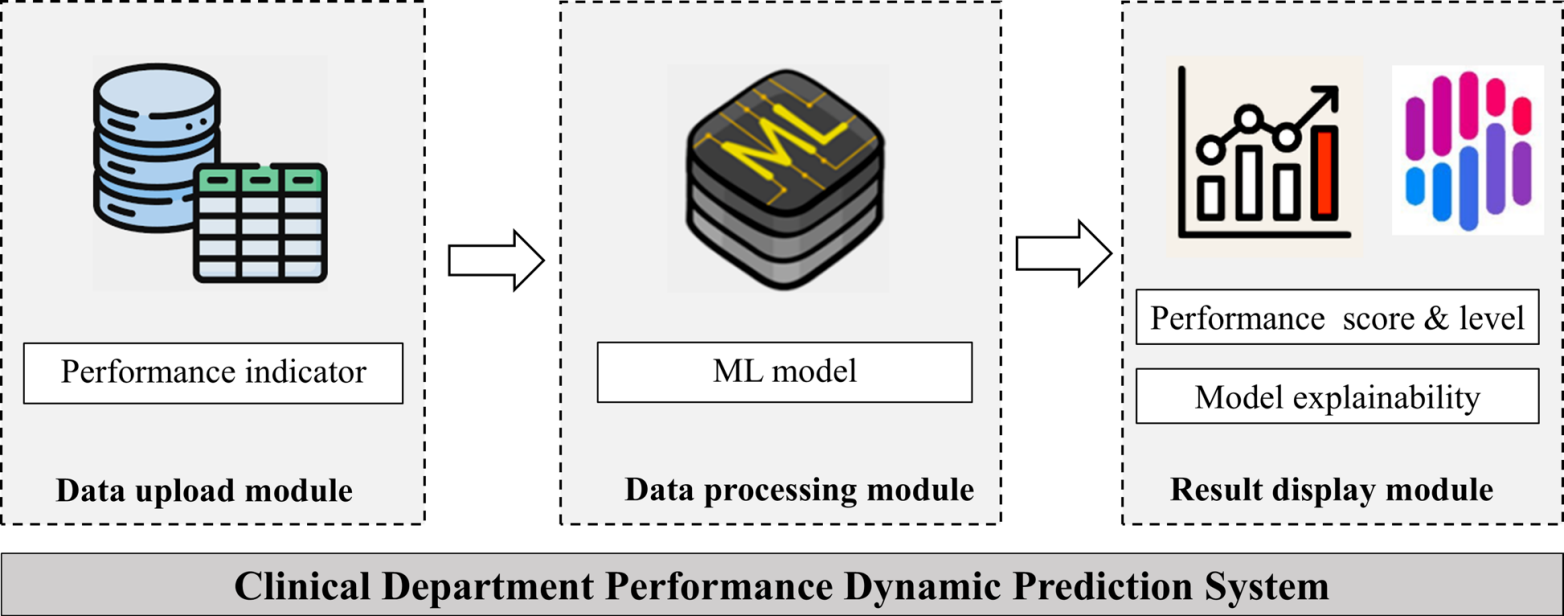
System module diagram

The Streamlit is an open-source Python library that helps users efficiently build and share data-driven applications, which has been widely used in various fields such as transportation, healthcare, and agriculture [35–39]. In this study, the web tool was developed based on the trained model for the prediction of clinical department performance scores. The main functional modules of the system included data uploading, model processing, and result presentation (Fig. 2). The system interface was designed intuitively, which greatly facilitated the dynamic prediction and analysis of clinical departments performance data.

## Results

### Model evaluation

**Fig. 3:**
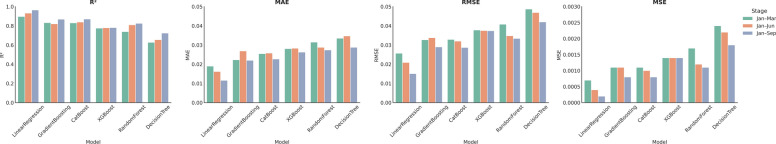
Model performance comparison across three cumulative time window

Figure 3 presented a comparison of six machine learning models trained under three cumulative time windows (January–March, January–June, January–September) in predicting annual performance score of clinical departments. The Linear Regression (LR) model consistently achieved the highest R^2^ values (0.8964, 0.9314, 0.9640) and the lowest MAE (0.0190, 0.0162, 0.0116), RMSE (0.0257, 0.0209, 0.0151) and MSE (0.0007, 0.0004, 0.0002) across all stages, demonstrating stable and accurate predictive performance. As the time window expanded, model performance improved progressively suggesting that incorporating additional monthly data enhanced the robustness of predictions. Thus, the LR model was selected as the optimal one and further integrated into the web tool for dynamic prediction system for clinical application.

## Model explainability

**Fig. 4:**
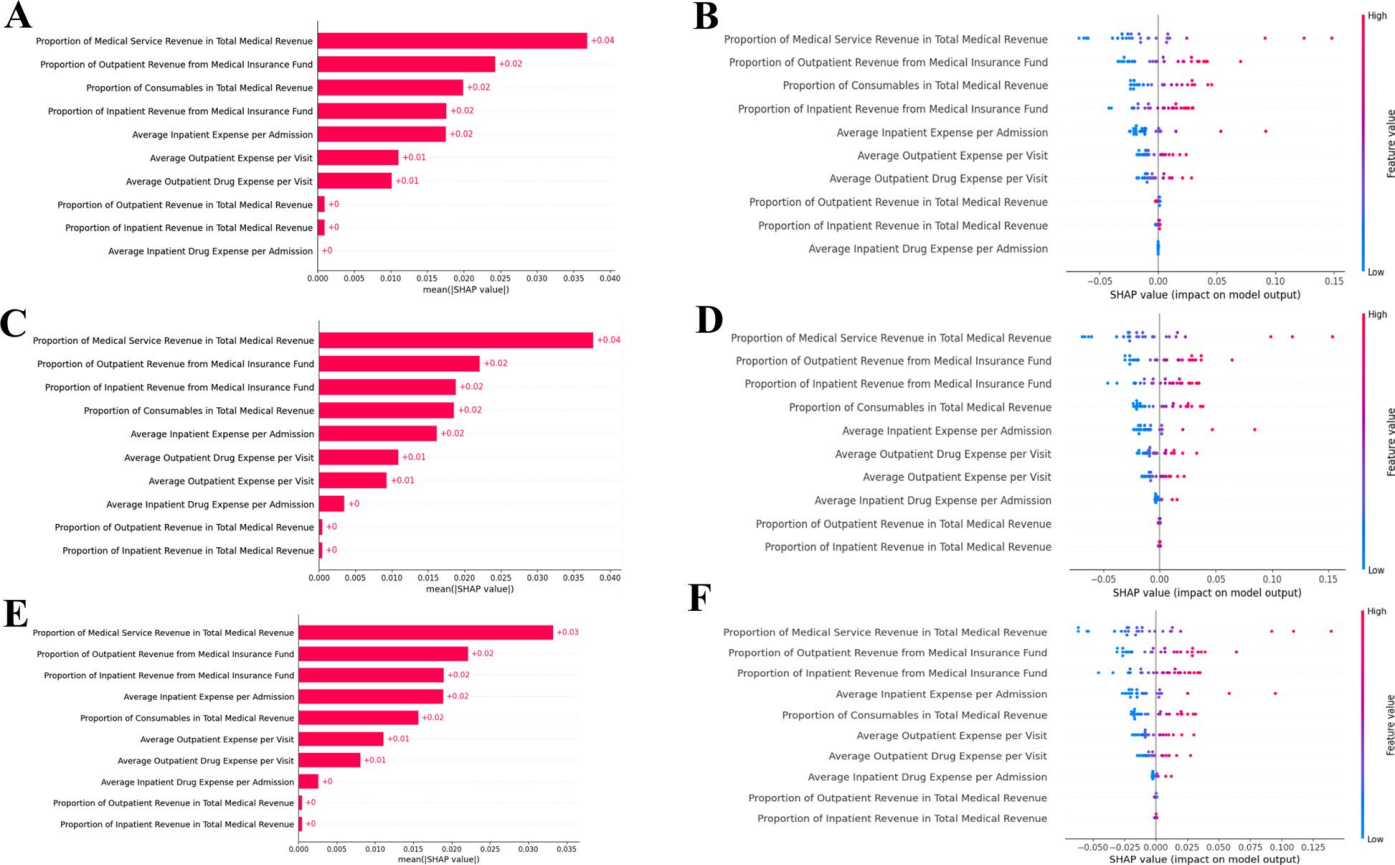
Global explainability of the model across temporal windows. Panels **A**,** B**: Jan–Mar; **C**, **D**: Jan–Jun; **E**, **F**: Jan–Sep. SHAP bar plot (A, C, E) and SHAP beeswarm plots (B, D, F)

To further interpret the predictive results of the model, SHAP-based explainability analysis was applied at both global and local levels. In terms of global explainability, the shap bar plots illustrated the feature importance to model predictions across different temporal windows (Fig. 4, Panels A, C, E). The top five contributing features were consistent across all stages, including Proportion of Medical Service Revenue in Total Medical Revenue, Proportion of Outpatient Revenue from Medical Insurance Fund, Proportion of Consumables in Total Medical Revenue, Proportion of Inpatient Revenue from Medical Insurance Fund, and Average Inpatient Expense per Admission. Although the top five features remained the same, their relative importance varied across windows. In addition, SHAP beeswarm plots (Fig. 4, Panels B, D, F) were used to visualize the distribution and direction of feature contributions for individual sample across different temporal windows. The comparison across different cumulative time windows further illustrates the stability of the top five contributing features, offering deep insights into the main drivers of clinical departmental performance.

**Fig. 5:**
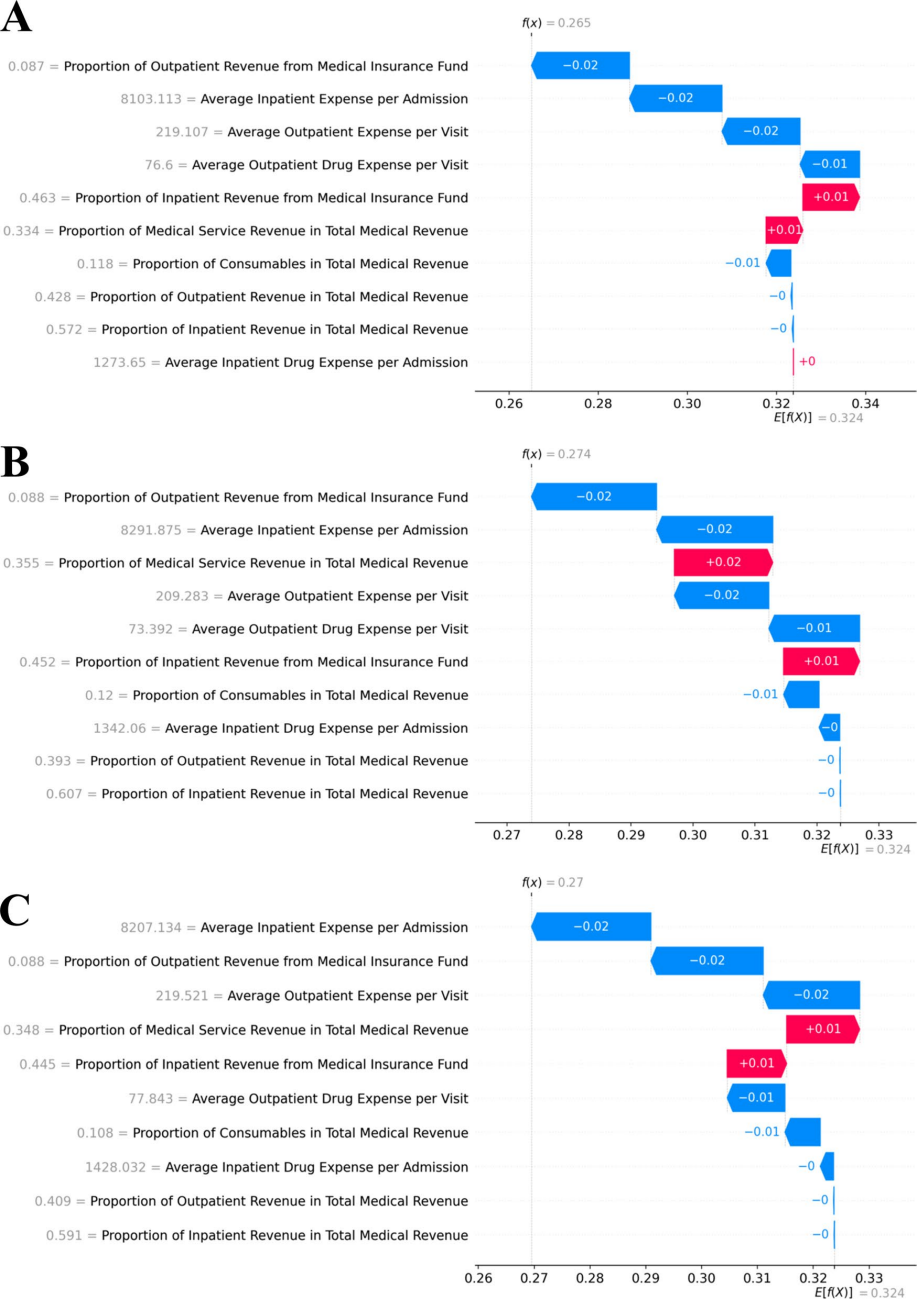
Local explainability of the model across temporal windows. Panels **A–C** show SHAP waterfall plots for Jan–Mar, Jan–Jun, and Jan–Sep, respectively

For example, in terms of local explainability, SHAP waterfall plots were used to illustrate the model’s prediction for the Department of Ophthalmology across three cumulative time windows (Fig. 5). The baseline prediction (expected value) was 0.324, and the predicted annual performance scores for the three windows were 0.265, 0.274, and 0.270, respectively. The SHAP waterfall plots revealed the contribution of each specific performance indicator value to the model’s prediction. In these plots, red bars indicated positive effects that increase the predicted annual performance score, whereas blue bars represent negative effects that decrease it, which enhanced the transparency and interpretability of the “black-box” model for deep insight at individual-level.

## System deployment

**Fig. 6:**
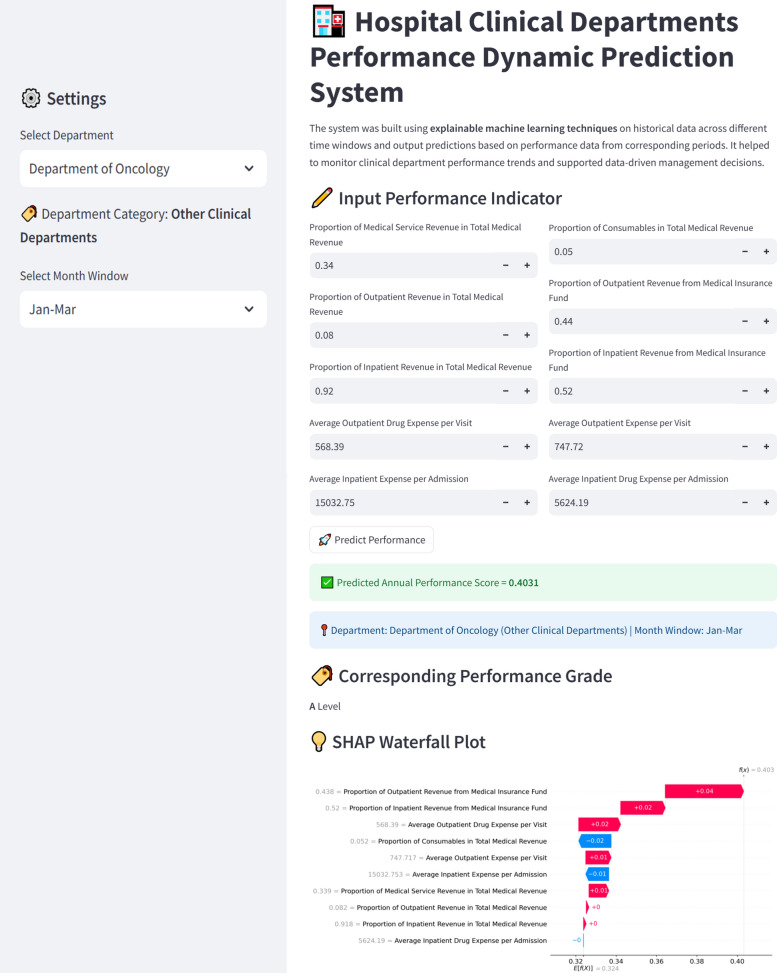
Interactive web interface for clinical department performance dynamic prediction system

The web tool, ClinDeptPredictor, was developed based on the trained model, accessible at https://clindeptpredictor-tool.streamlit.app/. The system supported multiple cumulative time windows, allowing to dynamically predict the annual performance score. The predicted score was then mapped to a performance level based on the quartile thresholds (Table 4). In addition, the system provided model interpretability through SHAP waterfall plots, revealing the contribution of each performance indicator to the predicted score at individual-level explanations. The interface was designed interactively, enabling convenient clinical department performance data input and real-time prediction result Fig. 5 Fig. 6.

**Table 4:** Mapping of annual total performance scores to performance levels

Range of annual total performance	Quartile	Performance levels
(0.209, 0.272)	Q1	D
(0.272, 0.304)	Q2	C
(0.304, 0.390)	Q3	B
(0.390, 0.469)	Q4	A

Quartiles are calculated based on the distribution of annual total performance scores for 2023. Q1 is the lowest quartile and Q4 the highest. Ranking A denotes the best performance and D the lowest

## Discussion

In this study, we developed a novel dynamic prediction framework for predicting annual performance scores of clinical departments in public hospitals using explainable machine learning. Six predictive models were constructed across three cumulative time windows (Jan–Mar, Jan–Jun, Jan–Sep), among which LR consistently demonstrated the best performance. To ensure model transparency, SHAP-based interpretability was applied to reveal feature contributions at both the global and individual levels. For practical utility, the interactive web tool, ClinDeptPredictor, was developed based on the trained model, enabling real-time prediction and individualized explanation for performance management.

Conventionally, hospital performance evaluation has predominantly relied on static annual indicators and traditional statistical methods. Prior studies have demonstrated that measures such as average length of stay, bed occupancy rate, mortality rate, and patient satisfaction are routinely used as fixed, retrospective indicators [40, 41]. Comprehensive evidence mapping further reveals that performance frameworks have been largely indicator-based and retrospective in nature [42, 43]. Methodologically, most performance studies have utilized traditional techniques, including data envelopment analysis (DEA), stochastic frontier analysis (SFA), balanced scorecard [44–46]. Although these approaches effectively measure productivity or efficiency, they inherently treat performance as a static output. As Pollack et al.[47] noted, prediction models in hospital settings are infrequently updated (i.e., static), and such static models may lead to misclassification compared to adaptive approaches, highlighting a critical limitation of current practices.

Against this backdrop, our framework advances the field in three key ways. First, we shift the paradigm from static assessment to dynamic prediction by introducing a progressive strategy that uses cumulative monthly data (e.g., Jan–Mar, Jan–Jun, and Jan–Sep) to predict end-of-year outcomes. The approach enables continuous monitoring and early intervention, an aspect rarely addressed in previous research. Second, we move from opaque models to interpretable machine learning by applying SHAP to offer transparent insights into how each indicator contributes to predictions across different temporal windows, thereby bridging the gap between black-box models and managerial decision-making. Third, we transition from generic metrics to policy-aligned indicators, with guidance by the government policy guidelines document and hospital operational practices to ensure multidimensional coverage. Collectively, the work transforms performance evaluation from a retrospective and static exercise into a proactive and interpretable process, indicating both methodological innovation and practical relevance.

The findings of the study mainly aligned with prior research. SHAP analysis revealed that the top 5 influential indicators of departmental performance were those related to revenue structure, insurance utilization, and cost control. Specifically, these included the proportion of medical service revenue, the proportion of revenue from medical insurance (outpatient and inpatient), the proportion of consumables, and the average inpatient expense (Fig. 4). This aligns with previous studies emphasizing revenue structure, insurance utilization, and cost control were critical drivers of departmental performance in public hospital performance [48–54]. Importantly, the consistency importance of top five performance indicators across different time windows suggests that the performance determinants are stable over time, supporting the validity of the proposed dynamic framework. Additionally, local-level explanations further demonstrated the direction and magnitude of each feature’s contribution to individual predictions, offering insights for managerial interventions.

The development of ClinDeptPredictor as a web system translates the methodological innovation into a practical decision-support tool. Hospital administrators can input current departmental data, obtain predicted annual performance scores and corresponding levels, and visualize the underlying drivers in real time. The integration of dynamic prediction and explainability supports evidence-based strategy adjustment before year-end and promotes proactive performance management. Ultimately, the proposed framework provides a novel approach for intelligent clinical departments performance monitoring and serves as a valuable reference for the future development of multi-center hospital performance management systems.

## Limitations

Several limitations of the study should be acknowledged. First, the study is conducted using dataset from a single public hospital within a specific national context and over a limited time period, which may constrain the generalizability and temporal robustness of the model. Second, the assignment of weights for performance indicators is based on the government policy guidelines document and the hospital’s operational practices, introducing a degree of subjectivity. Although the selection of specific performance indicators and its weight may differ across regions, the proposed dynamic framework for clinical departments performance prediction is generally applicable. With the availability of multi-center and longer-term performance data, future research could extend the framework to regional hospital networks, enabling dynamic prediction and data-driven performance management across multiple institutions.

## Conclusion

The study presents a novel dynamic framework based on explainable machine learning, which enables progressive early-stage prediction rather than end-of-year assessment, assisting administrators to monitor trends and implement timely interventions towards clinical departments performance. For practical utility, the framework is implemented as an interactive web tool, providing quantitative guidance to support performance management in hospital clinical departments. Overall, the work is particularly important in the context of China’s complex medical service landscape and offers valuable reference for other regions.

## Data Availability

Not applicable.

## Data availability

The dataset analyzed during the current study is not publicly available but can be obtained from the corresponding author upon reasonable request.
